# Digital Sequence Information between Benefit-Sharing and Open Data

**DOI:** 10.1093/jlb/lsac035

**Published:** 2022-11-22

**Authors:** Irma Klünker, Heiko Richter

**Affiliations:** Humboldt University of Berlin, Weizenbaum Institute for the Networked Society, Berlin, Germany; Max Planck Institute for Innovation and Competition, Munich, Germany

**Keywords:** Digital Sequence Information, Open Data, Genetic Information, Open Data Directive, Data Governance Act, Nagoya Protocol

## Abstract

Currently, parties to the Convention on Biological Diversity (CBD) are negotiating a strategic plan to save biodiversity. One crucial element of an agreement is the question of whether and how digital sequence information (DSI) is subject to access and benefit-sharing from the utilization of genetic resources, one main instrument of the CBD. In the EU, the Open Data Directive (ODD) of 2019 and the recently adopted Data Governance Act (DGA) already cover research data and to some extent DSI. An analysis of the ODD and the DGA throws a spotlight on the legal uncertainty of utilizing DSI and reveals systemic tensions between open data principles and benefit-sharing restrictions on non-commercial use. It also suggests that a future benefit-sharing mechanism for DSI should avoid distinguishing between commercial and non-commercial use upstream, but should instead favor a solution, which imposes benefit-sharing obligations further downstream or outside of the DSI life cycle.

## I. INTRODUCTION

In December 2022, the parties to the Convention on Biological Diversity (CBD)^1^ will come together at the Fifteenth Meeting of the Conference of the Parties (COP15) in Montreal to conclude negotiations on the Post-2020 Global Biodiversity Framework, a strategic plan to halt the rapid loss of biodiversity. Part of these negotiations is the question of whether and how digital sequence information (DSI) is subject to access and benefit-sharing (ABS) from the utilization of genetic resources, one main instrument of the CBD. In the complex negotiations with deep divisions between the Global South and the Global North, DSI could turn out to be a dealbreaker of the Post-2020 Global Biodiversity Framework.^2^

The Post-2020 Global Biodiversity Framework is not only crucial to combat biodiversity loss. A decision is also urgently needed because legal uncertainty is high under the status quo, as some countries have already adopted domestic measures regulating DSI, while others such as the EU are hesitant and await a decision on the international level.^3^ Six policy options are currently being negotiated, ranging from the full integration of DSI into domestic ABS measures to a levy on retail sales of genetic resources.^4^ The chosen policy decision will have a profound effect on research and development and especially basic research that generates and uses DSI, because the policy option could significantly alter the way in which DSI (often stored in publicly accessible databases) can be used.

In the EU, research data are already governed by the Open Data Directive (ODD)^5^ and the Data Governance Act (DGA).^6^ This article analyses the EU open data regulatory framework for research data and specifically DSI. The analysis of the EU open data regulatory framework throws a spotlight on the legal uncertainty of utilizing DSI and exposes the weaknesses of the current ABS system. This methodology allows it to generate implications, which can substantially feed into the ongoing negotiations on DSI and the COP15 in Montreal in December 2022. In particular, the findings suggest that from an open data perspective, caution should be exercised with regard to standard mutually agreed terms or licenses as a solution. Instead, the findings point towards a multilateral solution where benefit-sharing occurs later downstream. Additionally, the analysis reveals significant loopholes in the EU legislation and proposes changes accordingly. The article thus also generates new insights to readers who are interested in possible advancements of the horizontal regulation of public sector data in the EU. On a more abstract level, this article contributes to the fundamental discourse on the tension between efficient allocation and fair distribution of intangible resources and benefits,^7^ pointing to the infrastructural function of data for research-driven innovation. Although the analysis focuses on EU law, this allows it to exemplify the systemic tensions between upstream licenses on DSI to ensure benefit-sharing on the one hand and innovation from open data on the other.

This article is structured as follows: Section II contextualizes how the rapid advancements in genomics in the past 30 years have both presented a major challenge for ABS and at the same time sparked an open data culture reflected in recent EU legislation. Section III enquires more deeply into the ODD and exposes systemic tensions between open data principles and ABS licenses restricting the re-use of DSI. Section IV does the same for the DGA. Finally, Section V reconciles the findings by drawing implications for the negotiations on DSI at COP15 in Montreal in December 2022 and the EU open data regulatory framework.

## II. GENOMICS, DIGITAL SEQUENCE INFORMATION, AND THE EU LEGAL FRAMEWORK ON OPEN DATA

### II.A. The Genomics Revolution

In 1990, one year before the official negotiation of the CBD began, the Human Genome Project was launched, and after a little more than a decade of worldwide research and a race between the public consortium and a private initiative, first results of the deciphered human genome were published in 2001.^8^ The Human Genome Project is a success story for open data, as the project results would not have been published ahead of schedule if the genomic data had not been made openly available beforehand.^9^ Following an agreement called the Bermuda Principles in 1996,^10^ researchers of the public consortium shared their genomic data in the public domain within 24 hours of sequencing.^11^ The principles were widely adopted,^12^ and the sharing triggered a rapid advancement in genomics,^13^ which in turn spurred an open data practice unrivalled by other disciplines.^14^

The relevance of this scientific practice cannot be overestimated. From genomics, research has expanded along the pathway of gene expression to transcriptomics and proteomics, among many other research areas, which are referred to as the ‘omics’. Twenty years after the Human Genome Project, the leading role of omics with open data has allowed the development of a ground-breaking application of artificial intelligence:^15^ AlphaFold, a neural network that predicts the folded structure of proteins from their amino acid sequences, using data derived from public databases.^16^ Not the least, the rapid sharing of genomics data during the Covid-19 pandemic has played an important role in the development of vaccines and therapeutics.^17^

But the scope of the genomics data sharing practice extends beyond research and development; it has shaped open data policy.^18^ Given the fundamental societal importance of sharing genomics data, policy makers and legal scholars increasingly deal with the role of the legal framework to maximize benefits and to foster innovation in the life sciences for the general welfare. At the same time, the drastic change in technological possibilities presents a major problem for ABS: the question of whether and how ABS applies to the wealth of data generated from genetic resources. The following sections will explain how the two regulatory frameworks of benefit-sharing on the one hand and open data on the other have evolved in parallel with the genomics revolution, to then expand on how both frameworks could be reconciled.

### II.B. The Regulatory Framework for Genetic Resources and Ongoing Negotiations on Digital Sequence Information

#### 1. The Convention on Biological Diversity of 1992 and the Nagoya Protocol of 2010

In 1992, years of negotiations for a new international legal instrument to conserve the Earth’s rapidly decreasing biological diversity concluded in the CBD, which sets three objectives: the conservation of biological diversity, the sustainable use of its components, and the fair and equitable sharing of the benefits arising out of the utilization of genetic resources.^19^ It was a long way until 2010, when a mechanism to implement the objective of benefit-sharing was finally put into practice with the Nagoya Protocol.^20^ The Nagoya Protocol sets out a bilateral mechanism according to which potential users of genetic resources must obtain prior informed consent to access the genetic resource from the provider and then negotiate the conditions of utilization of the genetic resource and subsequent benefit-sharing in mutually agreed terms (MAT).^21^ As of today, the Nagoya Protocol counts 137 parties, but not Canada, Russia, or the US.^22^

In the EU, the Nagoya Protocol is implemented by the Nagoya Regulation.^23^ This Regulation establishes due diligence obligations of users of genetic resources within the EU,^24^ but it does not address questions of access to genetic resources, which remain within the competence of the Member States.^25^ In material scope, the biodiversity regulatory framework does not apply to human genetic resources,^26^ and certain plant genetic resources are exempt from the Nagoya Regulation because they are already subject to access and benefit-sharing under the International Treaty on Plant Genetic Resources.^27^

#### 2. The ‘Datafication’ of the Genome and the Status Quo of Digital Sequence Information

Following the Human Genome Project, research methods on genomics have significantly advanced. Moreover, not only has the amount of genomic data grown exponentially within these last two decades, but sequencing costs have decreased exponentially in the same period, even more quickly than Moore’s law as an indicator for a rapidly advancing technology.^28^ Accordingly, genomic data have become highly relevant in research and innovation. The Nagoya Protocol, however, does not explicitly address access and benefit-sharing of digitized data deriving from genetic resources.

Against this background, it is not surprising that the issue became pressing whether the intangible genomic data that stem from the sequencing of genetic resources, DSI, are also subject to access and benefit-sharing. This question is currently under debate between the parties^29^ and will be part of the negotiations on the Post-2020 Global Biodiversity Framework during COP15 in Montreal in December 2022. Clarifying if DSI falls under access and benefit-sharing is therefore crucial for the future of biodiversity.

An agreement is also urgently needed to provide research and innovation with legal certainty because some countries have already adopted domestic measures regulating DSI, but they follow very different approaches.^30^ For example, in South Africa, the term genetic resources includes information, and therefore benefit-sharing pertains to DSI as well.^31^ For access, users must obtain a specific permit, ie depending on whether the resource is intended for commercial or industrious exploitation or basic research abroad.^32^ The sharing of benefits is then only required for commercial or industrious exploitation.^33^ But to ensure that the resources or DSI are used for basic research only, the export permit for basic research is issued under the conditions that it is only used for non-commercial purposes and that transfer to a third party requires written consent of the issuing authority.^34^ Similarly, benefit-sharing in case of commercial or industrious exploitation is ensured contractually by stipulating that in case of a transfer to third parties, the third party must be bound equally by these terms.^35^ A distinction between non-commercial and commercial use and the obligation to impose the same obligations on subsequent users is not uncommon in access and benefit-sharing regulation.^36^ They originate in Article 8(a) Nagoya Protocol, which stipulates that research, which contributes to the conservation and sustainable use of biological diversity should be promoted through simplified measures on access for non-commercial research purposes.^37^ This way, DSI may be subject to contractual restrictions on the type of use as well as on subsequent benefit-sharing to ensure that benefits are shared according to the domestic legislation of the provider country. Thus, access and benefit-sharing of DSI can already be a reality in the EU: According to a guidance document of the European Commission, DSI ‘could be considered to be out of scope of the ABS Regulation’, but ‘the use or publication of such data might be covered by conditions set in the mutually agreed terms, which should be respected’.^38^ In other words, while the due diligence obligations imposed by the EU Nagoya Regulation in Article 4 may not apply to DSI, users of DSI can be bound contractually by MAT.

However, other countries have implemented very different approaches to domestic measures on DSI.^39^ Legislation in Brazil, for example, requires users of DSI, or *in silico* genetic heritage under Brazilian law, to register their use through an online system, but benefit-sharing is only required if the use of DSI is commercialized.^40^ Still, the South African example illustrates how domestic measures can translate into contractual restrictions on the re-use of DSI. Such restrictions or licenses could also be part of some of the policy options currently negotiated.

In view of the negotiations at COP15, a working group has prepared six policy options for DSI:^41^

Option 0: Status quo.

Option 1: DSI fully integrated into domestic ABS measures.

Option 2: Standard MAT or licenses.

Option 3: Payments and contributions to a multilateral fund.

Option 4: Technical and scientific capacity and cooperation.

Option 5: No benefit-sharing from DSI.

Option 6: A one per cent levy on retail sales of genetic resources.

The broad range of policy options displays the preliminary stage of the negotiations and reveals the high complexity of establishing a new framework for DSI. It should be noted that option 6 was added later on.^42^ Option 6 is similar to option 3 in that it proposes a multilateral fund. Options 3, 4, 6 and the standard MAT on an international level could build on a global multilateral benefit-sharing mechanism^43^ as envisaged in Article 10 Nagoya Protocol.^44^ Out of the six policy options, three are the most likely to represent both a feasible solution and a realistic political compromise: standard licenses for using the data (option 2), access fees to databases or a micro-levy on laboratory equipment to contribute to a multilateral fund (option 3), or a levy on products of genetic resources (option 6). Option 1 does not seem to be a feasible solution because of the evident high transaction costs. Options 4 and 5 do not seem politically realistic, because they do not generate monetary benefits.

However, the policy options currently negotiated are not only very different from one another, but each option comes with a range of possibilities regarding its implementation. The draft recommendation from the latest preparatory meeting in Nairobi also contains a proposal for a hybrid solution, which could be a combination of options 1 and 2 or a combination of international MAT and the establishment of a multilateral fund.^45^ Such a hybrid solution could also include option 4. The following analysis will assess option 2 from an open data point of view, but some implications can also be drawn for options 3 and 6.

What exactly is DSI? The material scope of DSI is still unclear. So far, the term DSI has only been used as a placeholder in policy debates.^46^ From a biosciences point of view, the genome refers to the entire genetic information of an organism that is stored in the DNA, coded into a sequence of four different nucleotides represented by the letters A, T, C, G. In the genetic flow of information from the genome to a working protein,^47^ the required information is transcribed into RNA, which is very similar to DNA, and then translated into an amino acid sequence folding into a protein. Genomics is mainly concerned with DNA and RNA sequence data, which is nucleotide sequence data. Along the flow of genetic information, other omics research fields developed—from transcriptomics to proteomics, but also epigenomics for hereditary information not stored in the nucleotide sequence and metabolomics for the study of molecules involved in the metabolism. All of the data and information resulting from these omics could potentially be regarded as DSI, and the exact definition is still open for debate in the COP15 negotiations in Montreal.^48^ This article focuses on nucleotide sequence data, which is the common ground of the discussed material scopes and which in practical terms is also the basis of genomics research. However, one should keep in mind that DSI could be of a much broader scope.

### II.C. The Regulatory Framework for Public Research Data in the EU

DSI concerns data that stem from genetic resources. This origin is the reason why its coverage under the access and benefit-sharing principles of the Nagoya Protocol is vividly discussed. But at the same time, it must not be overlooked that DSI also falls within the broader category of research data, the generation of which is largely funded by the public. Considering this link to the public sector and public funding, public sector information legislation is relevant for DSI as well. The EU has established a legal framework for public sector information in the last two decades, which today also includes publicly funded research data—and arguably also the re-use of DSI. Two legislative acts are particularly relevant: the ODD and the DGA. As an EU Directive, the ODD addresses Member States, which then transpose the provisions set out in the Directive into national legislation. In contrast, the DGA is an EU regulation, which means that its provisions apply directly to its addressees.

In substance, and unlike the Nagoya Protocol, the ODD and the DGA do not apply to the tangible genetic resource as such, but only to the digitized information as research data that may relate to it. Therefore, their relevant addressee is not the holder of the tangible genetic resource, but the public research institution or publicly funded researcher who holds the intangible DSI subsequent to genome sequencing, which was enabled by access to the tangible genetic resource. While the ODD stipulates open data principles for re-use and therefore aims for a maximum degree of data dissemination, the DGA complements the ODD with a legal framework for the selective sharing of public sector data, according to which re-use is allowed only in certain cases for specific purposes. The following sections will enquire deeper into the ODD (under III) and the DGA (under IV) to discuss their interrelation with ABS and how open data principles can contribute to the current debate on policy options for DSI.

## III. THE EU OPEN DATA DIRECTIVE AND DIGITAL SEQUENCE INFORMATION

### III.A. Development and Goal of the Open Data Directive

While the sheer need for cooperation has sparked strong open data traditions in the genomics research community, open research data has escaped EU^49^ regulation for a long time. Only since 2019 have open research data been regulated by the ODD. The ODD provides for a harmonized set of rules for the re-use of data^50^ held by public sector bodies (PSBs) and public undertakings within the EU, but it also contains specialized rules for research data. The ODD is a recast of the Public Sector Information (PSI) Directive, which dates back to 2003.^51^ However, until its recast as the ODD in 2019, the PSI Directive explicitly excluded the application from educational and research establishments, which is research data.^52^ Already in 2013, discussions took place as to whether educational and research establishments should be included in the amendment, but the idea was rejected because of lack of feasibility.^53^ Since then, however, the EU has implemented policies advocating open data, including open access policies in EU-funded research.^54^ With the recast of the PSI Directive in 2019, research data was finally included within the scope of the Directive.

The goal of the ODD is to allow for the re-use of publicly funded data for non-commercial and commercial purposes and thereby foster innovation.^55^ The extension to research data aims to exploit the exponentially growing research data beyond the scientific community.^56^ Moreover, the ODD aims to foster future innovation and names artificial intelligence as one such field.^57^

### III.B. The Open Data Directive and Research Data

The ODD addresses research data in its Article 10, but with two important limitations: First, it addresses only publicly funded research data. Second, the provision only regulates the conditions for re-use of such data without mandating access to it. This focus on re-use stems from the underlying rationale of the ODD as a re-use regime,^58^ and it explains the twofold conceptualization of Article 10 ODD, which is reflected in its Sections 1 and 2.

Article 10(1) ODD is a general and non-enforceable ‘political obligation’, which calls on the Member States to adopt ‘national policies and relevant actions aiming at making publicly funded research data openly available’. Such open access policies should follow the principle of ‘open by default’. Moreover, they should abide by the FAIR principles, an acronym which stands for research data being findable, accessible, interoperable, and re-usable.^59^ The FAIR principles were proposed by a group of stakeholders from academia, industry, funding agencies, and scholarly publishers.^60^

In contrast, Article 10(2) ODD sets out concrete substantive conditions regarding the re-use of publicly funded research data, which the Member States must implement. The provision stipulates that:

research data shall be re-usable for commercial or non-commercial purposes in accordance with Chapters III and IV, insofar as they are publicly funded and researchers, research performing organizations or research funding organizations have already made them publicly available through an institutional or subject-based repository. In that context, legitimate commercial interests, knowledge transfer activities and pre-existing intellectual property rights shall be taken into account.

In the following, we will discuss how these rules affect the re-use of genomic data in general. We will focus on the specific scope of application of the ODD on DSI (III.C) and discuss the consequences for the licensing of re-using such data (III.D). We conclude with a summary of the systemic tensions between open data and licenses in benefit-sharing (III.E).

### III.C. Scope of Application and Relevance for Digital Sequence Information

#### 1. Documents in a Digital Form

Generally, the ODD applies to a broad range of information and data that is held by PSBs and public undertakings. For the covered subject matter, it uses the old-fashioned term ‘documents’, defined as ‘any content whatever its medium (paper or electronic form or as a sound, visual or audiovisual recording)’ and ‘any part of such content’.^61^ This definition is intentionally framed broadly to accommodate new technologies^62^—a feature, which is especially relevant in the rapidly advancing genomics and biotechnology landscape.^63^

Article 2(9) ODD specifies research data as a fraction of all data covered by the ODD, and to which Article 10 ODD applies. It defines research data as:

documents in a digital form, other than scientific publications, which are collected or produced in the course of scientific research activities and are used as evidence in the research process, or are commonly accepted in the research community as necessary to validate research findings and results.

The ODD provides a list of examples for research data, which includes ‘statistics, results of experiments, measurements, observations resulting from fieldwork, survey results, interview recordings and images. It also includes meta-data, specifications, and other digital objects.’^64^ Due to this broad definition, a wide range of research data in the genomics research field may potentially^65^ fall under the provisions of the ODD. Beginning from the sequencing of genomes, typical data generated and used in genomics are the raw sequencing output of nucleic acid sequences (DNA and RNA), meaning a file with sequences of A, T, C, G in the case of DNA (a file with sequences of A, U, C, G in the case of RNA^66^).^67^ The ODD would also cover the annotated sequences, ie sequences which have been aligned with similar sequences such as a reference genome and which have genes mapped to the genome. These are typical examples of data in genomics, which can be considered research data within the meaning of the ODD, but of course research data can encompass many more data. In practice, different file formats are used for genomic data, such as the FASTA format for sequence data or the BED for annotated sequences. Overall, genomics has relatively standardized data formats, another legacy of the Human Genome Project.^68^ While a definition of the term DSI is still subject to ongoing negotiations, the narrowest scope of DSI discussed is nucleotide sequence data.^69^ It is important to note that the term research data also covers genomic data from humans, which are not, however, considered as DSI. In that case, data protection has to be taken into account.^70^

The ODD does not apply to computer programs but leaves it to the Member States to extend the application to them.^71^ Such extension of the ODD’s scope can become relevant to code, which is usually provided in specific sections that follow the main article in genomics research publications. The code is often made available in repositories such as Github or Zenodo. However, if Member States were to extend the application to code, the interplay with copyright rules would need to be defined precisely.

#### 2. Data Other than Scientific Publications

The ODD excludes scientific publications from the definition of research data.^72^ This criterion distinguishes research data from copyrighted material.^73^ Such explicit delineation is necessary because the ODD has so far only generally exempted documents for which third parties hold intellectual property rights,^74^ but not where the addressees of its obligations—such as the researcher—hold copyrights themselves.^75^

In substance, the exclusion of scientific publications aims to provide flexibility and preserve ‘individual research actions’.^76^ As a consequence, it leaves ample discretion to the researcher in terms of whether scientific information is to be qualified as research data, as part of a scientific article, or as a data paper.^77^ This rather formalistic definition reflects the fundamental right of the freedom of scientific research.^78^ Importantly, it reveals the basic conceptualization of the ODD as a tool for increasing the dissemination of research data while still accounting for the standards of the research community to set incentives for making research data publicly available. It has been shown that both communal norms of science and mandatory rules to deposit data play an important role in genomic data sharing.^79^

Looking at scientific publications in genomics, the main scientific article is usually followed by a section on data availability with the accession numbers, followed by a section on code availability and sometimes also extended data.^80^ Therefore, the referenced data qualifies as research data within the meaning of the ODD,^81^ while the main article does not.^82^

#### 3. Generated during Research Activities and Used as Evidence or for Validation

Article 2(9) ODD also requires that the research data are ‘collected or produced in the course of scientific research activities and are used as evidence in the research process, or are commonly accepted in the research community as necessary to validate research findings and results’. Without this criterion, documents in a digital form other than scientific data would effectively include all data produced during research (eg notes of the researcher, communication, or lab instructions, except for scientific publications). Again, the ODD relies on the research community to determine which data are necessary to validate research results. In genomics, nucleotide sequence data are crucial to determine and validate genomic functions. For this reason, the research community has long established the standard of providing nucleotide sequence data in repositories alongside a publication.

#### 4. Public Funding

In general, the ODD only applies to data held by PSBs or public undertakings. However, there is an exception for research data: For the ODD to apply, it is irrelevant by whom the research data are generated and held (be it in public research institutions or private companies^83^) as long as the research data are publicly funded.^84^ This funding requirement refers solely to the data themselves, not to the funding of the researcher or research organization.^85^ In practice, research data produced in universities or other big research organizations^86^ are usually publicly funded.

In scientific research, public-private co-funded research data is of particular relevance.^87^ In this regard, Recital 28 ODD states that: ‘certain obligations stemming from this Directive should be extended to research data resulting from scientific research activities subsidized by public funding or co-funded by public and private-sector entities’. However, it remains open what this exactly means. Closely related to public-private partnerships are legitimate commercial interests, which Member States must account for in the implementation.^88^ Therefore, despite the inclusion of partly publicly funded research data, the ODD may eventually not apply in public-private partnerships.

Regardless of the applicability of the ODD to data generated in public-private research partnerships, contractual obligations and conventions of the research community further affect dissemination practice. If the parties to a public-private partnership agree that the research results are to be published in an article, then the underlying data would of course have to adhere to the journal publishing standards. In genomics research, this would mean that at least the nucleotide sequence data would have to be made publicly available with the publication. Again, this demonstrates the impact of research community standards on the openness of research data.

#### 5. Repositories

Finally, the ODD requires that the research data be made publicly available through an institutional or subject-based repository by researchers, research performing organizations, or research funding organizations.^89^ Vice versa: If the research data have not yet been made available by such means, the ODD does not apply. At the same time, the ODD leaves it to the Member States to also include publication of the research data ‘through other data infrastructures than repositories, through open access publications, as an attached file to an article, a data paper or a paper in a data journal’.^90^

For genomics data, a diverse range of public subject-based repositories exists.^91^ Databases differ in the type of data they store, from general nucleotide sequence data to specialized databases such as a database related to SARS-CoV-2^92^ specifically. There are also databases that store raw sequences (primary databases) and others that store curated data (secondary databases). Despite the diverse range of repositories in genomics, the research community has established standards to centralize data in public databases, again a legacy of the Human Genome Project.^93^ The most important standard relates to the nucleic acid sequence data underlying a research paper, which in most cases must be uploaded to primary databases, usually to one of the three databases of the International Nucleotide Sequence Database Collaboration (INSDC) or the Chinese Genome Sequence Archive.^94^ The three databases of the INSDC are GenBank (operated by the US-American National Center for Biotechnology Information), the European Nucleotide Archive (European Bioinformatics Institute, UK), and the DNA Data Bank of Japan (National Institute of Genetics, Japan). These three databases exchange the submitted nucleotide sequence data, and curated data from these databases are then taken for secondary databases such as the UCSC Genome Browser, to which researchers cannot submit data directly. The importance of the INSDC repositories as core databases in genomics research cannot be underestimated.^95^

Institutional repositories, on the other hand, were established as a possibility for affiliates of the institution to publish immediately, long-term, and barrier-free, and as an alternative to the traditional publishing system. Many universities have their own repository and offer the possibility to make research data available. However, regarding genomics data, institutional repositories are not of high relevance.

Finally, the ODD requires that the research data is made available by researchers, research performing organizations, or research funding organizations.^96^ The Directive lacks definitions for these terms. Research performing organizations or research funding organizations under the meaning of the ODD are not necessarily organized as PSBs.^97^ Recital 28 ODD acknowledges that research performing organizations could be organized as PSBs or public undertakings, but also states that the ODD applies ‘to such hybrid organizations only in their capacity as research performing organizations and to their research data’. Yet private companies can also be research performing organizations as long as the research data are publicly funded.^98^

### III.D. Requirements for Licences on Re-Use as a Consequence

#### 1. Overview

If the data in question meet all previously mentioned requirements, the provisions of the ODD for re-using the research data are applicable. In particular, Article 10(2) ODD mandates that such research data must be re-usable for both commercial and non-commercial purposes. The specific modalities of re-use are set out in Chapters III and IV of the ODD.^99^ Most important for research data sharing practice are the provisions on licensing under Article 8, which are discussed in the following.

#### 2. Standard under the Open Data Directive

Because licenses on DSI are currently negotiated as a possible solution under policy option 2 (standard MAT), the provisions on conditions or licenses in Article 8 ODD can provide insights on the effects of such licenses from an open data perspective. From this perspective, licenses that restrict re-use may have legitimate reasons, but they stand in tension with the general principle of the ODD to maximize the dissemination and re-use of such data.^100^ Therefore, the ODD stipulates the open data principle on the one hand, while allowing this principle to be limited by licenses subject to justification (which national courts can review) on the other hand. Article 8(1) ODD sets the limit for restrictions via licensing:

The re-use of documents shall not be subject to conditions, unless such conditions are objective, proportionate, non-discriminatory and justified on grounds of a public interest objective. When re-use is subject to conditions, those conditions shall not unnecessarily restrict possibilities for re-use and shall not be used to restrict competition.

Licenses that address the dissemination of scientific research are very common to both scientific publications and research data.^101^ In practice, standard licenses^102^ significantly decrease transaction costs and are frequently used. Most prominently, the Creative Commons (CC) licenses provide a modular system of licenses. The modules are attribution (BY), non-commercial (NC), no derivatives (ND), and share-alike (SA), and they can be stacked together.^103^ Therefore, the openness of CC licenses varies from the public domain (CC0) to mostly closed (CC-BY-NC-ND). For example, under EU grants, the CC0 and CC-BY licenses are recommended.^104^ This example also demonstrates the impact of funders on the openness of research outputs.^105^

As for the requirements of the ODD, it has been intensively discussed to what extent licensing under CC modules complies with Article 8(1) ODD and is therefore legitimate. The most problematic is the NC-commercial module.^106^ While the ODD allows PSBs to differentiate between commercial and non-commercial re-uses with regard to royalties and further conditions,^107^ CC-NC licenses prohibit commercial re-use *per se*. Such restrictive licenses would run counter to the overarching goal of the ODD to open public data for commercial re-uses and therefore for privately created innovation. According to this view, Article 8(1) renders non-commercial clauses void.^108^ At first glance, this holds true for research data as well, because Article 10(2) ODD also stipulates that research data can be re-used for both commercial and non-commercial purposes.

#### 3. Relevance for Digital Sequence Information

In a scenario of the current bilateral ABS mechanism where the domestic legislation of a provider country mandates MAT that restrict commercial re-use of DSI to ensure benefit-sharing, researchers may not be able to publish their work according to the common scientific practice in genomics: If a researcher in the genomics field wants to publish research results, the publishers require the researcher to make the sequence data available in a data repository upon publication of the article. Regarding nucleotide sequence data, the accepted repositories are usually the three databases of the INSDC.^109^ However, the INSDC databases do not accept any conditions (including non-commercial) on the re-use of the uploaded data sets.^110^ There are other repositories that restrict the transfer of data to third parties or use CC licenses, eg the GISAID database for data from influenza viruses and the coronavirus causing COVID-19 or the NBN atlas, a collection of biodiversity data.^111^ However, these databases are not always accepted for journal publications, especially regarding nucleotide sequence data.

As such, in the current bilateral ABS mechanism, MAT provisions can already clash with the current scientific practice.^112^ This adds to the legal uncertainty and could disincentivize researchers from generating DSI from provider countries with such requirements. As the ODD builds upon and often refers to the scientific practice and requires the research data to be made publicly available for their application, it remains toothless against these tensions. However, as databases other than the INSDC allow restrictions on the re-use of data, and as the ODD also applies to institutional repositories and, depending on the Member State implementation, to data journals, it is worth exploring the application of the ODD to these cases for DSI. The analysis exemplifies the systemic tensions between MAT licenses and open data for innovation, reveals loopholes in the EU Open Data Framework, and guides the following analysis of currently negotiated policy options for a future regulatory framework for DSI.

There are four alternatives for researchers to publish their data other than via the established subject-based repositories required for journal publications. The first two are alternatives under the existing database landscape; the second two present potential future databases adjusted to MAT licenses. First, researchers could also publish the data in institutional (as opposed to subject-based) repositories, which would also fall under the scope of the ODD. Second, the Member States may choose to extend the application of the ODD ‘to research data made publicly available through other data infrastructures than repositories, through open access publications, as an attached file to an article, a data paper or a paper in a data journal’.^113^ In fact, data journals present researchers with an attractive alternative to repositories.^114^ It remains to be seen if the majority of such journals would also decline to publish datasets that are restricted to non-commercial re-use only.^115^ Third, one could also think of establishing other subject-based repositories for DSI data, which adhere to the legal requirements of the Nagoya Protocol.^116^ Or fourth, the INSDC databases could potentially implement MAT licenses.^117^

It becomes clear that in any of these cases, the data could be subject to the ODD, which may then indeed lead to a clash with Article 8(1) ODD. So ultimately the crucial question for making DSI publicly available is whether Article 8(1) ODD strictly prohibits non-commercial publication and would thereby prevent the presented alternative publishing means. This question is also highly relevant for the ongoing negotiations of policy options because option 2 of standard MAT or licenses discusses ‘obligations for commercial and non-commercial uses of a particular DSI’,^118^ and the ‘distinction between commercial and non-commercial use of DSI’ is one criterion for the assessment of the policy options.^119^ We propose three reasons in the following why, in our view, the ODD permits restrictions to non-commercial re-use of DSI.

First, the ODD leaves open how to treat merely relative restrictions that a third party has contractually imposed on the holder of research data regarding re-use. Good reasons suggest that any such contractual restrictions must prevail and may therefore amount to legitimate non-commercial restrictions regarding re-use of such data. The ODD does not aim to overrule legitimate interests of third parties in attaching strings to the re-use of data that is provided to the public sector and then (possibly) passed on to commercial re-users.^120^ Therefore, Article 8(1) is only applicable to restrictions, which the PSB imposes in addition to the ones to which it is bound vis-a-vis the third party.

Second, the ODD aims to promote the dissemination of data, but not at the expense of reducing incentives for innovation, meaning for the generation of data.^121^ Intellectual property rights—especially industrial property rights—could provide such incentives, and the ODD clearly states that it does not affect these rights.^122^ Furthermore, incentives to create research data would also be undermined if the ODD would affect public-private partnerships. Accordingly, the ODD requires Member States to account for legitimate commercial interests when implementing the provisions on research data.^123^ One example of such interests given by the ODD are trade secrets.^124^ While DSI is usually not kept as a trade secret,^125^ the interests of the provider of genetic resources in benefit-sharing are similar to the interests of a company in its intellectual property and other commercial interests in a public-private partnership. In sum, it becomes evident that the ODD itself already contains the rationale for not being interpreted in a way, which would run counter to MAT-induced restrictions.

Third, an additional argument for why Article 8(1) would ultimately allow for non-commercial restrictions in the case of DSI stems from drawing a functional parallel to the ODD’s treatment of personal data. As has been shown, the ODD aims not to interfere with incentives for the generation of data, while at the same time promoting the most open possible dissemination of data. This rationale is reflected in provisions of the ODD that concern personal data. In fact, research data can be personal data and thus be subject to the provisions of the EU General Data Protection Regulation (GDPR). The ODD clarifies that it is without prejudice to the GDPR;^126^ in particular, the ODD does not apply if the re-use of data is incompatible with data protection.^127^ However, if the re-use of data is not altogether incompatible with data protection, conditions within the meaning of Article 8(1) ODD may be imposed to comply with data protection requirements.^128^ Human genetic resources are not regulated by the Nagoya Protocol. However, DSI and human genetic resources are similar in that they are, though for different reasons, governed by an additional regulatory framework restricting the dissemination of research data. There has been a similar development in genomics data sharing policy from non-sharing for privacy reasons to alternate data sharing plans, which meet privacy requirements.^129^ Repositories for biological data with a controlled-access model are often specialized in personal data.^130^ The reasoning of ‘as open as possible, as closed as necessary’^131^ should therefore apply in the case of DSI.

Thus, all three considerations point towards an interpretation of Article 8(1) ODD that allows a restriction to non-commercial re-use in the case of DSI. The problem, however, is that none of them is explicitly spelled out by the ODD. Also, the legislature did not have DSI in mind as an application case of the ODD. The unclear wording of the ODD thus adds to the legal uncertainties of DSI.

### III.E. Systemic Tensions between Open Data and Licences in Benefit-Sharing

In sum, the analysis of the ODD reveals that genomics data as research data can indeed fall within the scope of the Directive. Therefore, the ODD is highly relevant for DSI. At the same time, the Directive only provides a general data sharing regime and leaves many details to be determined by the research community. While the genomics research community has an established culture of open data, its current practice is at odds with restrictions to non-commercial use in MAT. Depending on the domestic legislation on ABS, such restrictions are sometimes already required, but because they are also discussed as part of policy option 2 on standard MAT or licenses, this is also highly relevant for the ongoing negotiations on an international benefit-sharing mechanism that includes DSI.

It is crucial to understand that in the EU, the viability and perspective of such licenses significantly depend on the interpretation of the ODD. However, the described situation illustrates the systemic tensions between open data and benefit-sharing: On the one hand, the wording of Article 10(2) ODD reflects the general principle of the ODD that there should be no distinction between commercial and non-commercial re-use to foster innovation.^132^ On the other hand, in the current bilateral ABS system, a distinction between commercial and non-commercial use is often necessary to protect the provider’s interest in benefit-sharing if the purpose of use changes.^133^ The ODD recognizes that in some cases, there are limits to open data, which is reflected in its policy of ‘as open as possible, as closed as necessary’ in Article 10(1) ODD. It must be noted that innovation and benefit-sharing in themselves are not inherently antagonistic. On the contrary: Predictable monetary benefits are the first policy goal of the currently negotiated policy options for DSI.^134^ But to generate monetary benefits from DSI in the first place, eg by developing and selling a new pharmaceutical product, DSI has to be *used commercially*. A distinction between non-commercial and commercial seems especially misplaced in biotechnology, where the boundaries between basic and applied research are often blurred.^135^

We have argued that the ODD is to be interpreted in a way that it would allow for restrictions to non-commercial re-use if required for benefit-sharing. This comes, however, with three caveats – first, the proposed interpretation is not made sufficiently clear in the text of the ODD. This legal uncertainty within the ODD must be eliminated, and below we make some more concrete suggestions as to how this can be achieved (see V.B). Second, any amendment of the ODD must be seen in conjunction with the recently adopted DGA, which complements the ODD in the EU open data regulatory framework. We address this interplay of the ODD and the DGA and its relevance for DSI in the next section. The third caveat is that the ‘ODD-intrinsic’ approach is only a second-best solution. Restrictions on non-commercial re-use run against open data principles as well as against the scientific community’s open access practice. The root of the problem is the extension of the bilateral access and benefit-sharing mechanism to DSI, which already takes place in practice. The systemic tension between benefit-sharing and open access can only be dissolved within the system of benefit-sharing itself and cannot be remedied by merely amending the general regulatory framework on open data. The analysis of the ODD therefore stresses the need for an international agreement on how to address DSI. We discuss these tensions in more detail in the context of the currently negotiated policy options under Section V.

## IV. THE EU DATA GOVERNANCE ACT AND DIGITAL SEQUENCE INFORMATION

### IV.A. The Data Governance Act as a Complementary Tool to the Open Data Directive

On 16 May 2022, the DGA was adopted in the form of an EU Regulation. The DGA complements the scope and function of the ODD in that it applies to data, which fall outside the scope of the ODD because their re-use is conditional on the rights of others, in particular commercial and statistical confidentiality, intellectual property protection, or personal data.^136^

Similar to the ODD, the goal of the DGA is to facilitate the use of such data for research and innovation by private and public entities.^137^ In substance, the DGA stipulates mandatory conditions for commercial and non-commercial re-use of data that fall under its scope.^138^ Most importantly these are: first, the prohibition of exclusive arrangements;^139^ second, legal, technical, and organizational conditions for re-use; third, conditions for the fees charged for re-use.^140^ In this regard, the DGA was heavily inspired by the ODD. However, there is a striking difference as to the basic regulatory mechanism: The DGA does not create an obligation for PSBs to allow re-use of the data they hold.^141^ Only if the PSB has allowed re-use do the conditions for re-use have to fulfil the requirements of the DGA.^142^

### IV.B. Relevance for DSI

As with the ODD, the legislature did not have DSI in mind when designing the DGA. Rather, the EU data policy debate focused on data held by statistical offices as well as health, social, and economic data.^143^ However, the analysis of the DGA with regard to DSI does not only reveal a major conceptual flaw of the DGA whose significance extends beyond the application to DSI. The principles of the DGA are also relevant for licenses as one of the currently negotiated policy options for DSI because the DGA’s tools could be used to enable the dissemination of DSI for follow-on innovation in that policy option.

The major conceptual flaw is revealed by enquiring into the applicability of the DGA to DSI: Article 3(1) DGA exhaustively enumerates the cases in which the DGA applies. The data in question must involve commercial and statistical confidentiality, intellectual property protection, or personal data. However, under the current bilateral benefit-sharing mechanism, DSI falls in no such category, because depending on the domestic legislation of the provider country, the research institution is only bound by obligations, which have been imposed either by means of bilateral contractual limitation on re-use (MAT) or unilaterally imposed restrictions (eg by administrational permission). Obligations, which aim to ensure benefit-sharing, do not constitute an IP right.^144^ Against the background of the DGA’s clear wording, it would appear too far-fetched to interpret Article 3(1) DGA so broadly that data that are subject to mere contractual restrictions—such as MAT—would fall under the scope of the DGA. Such a reading would ignore that safeguards in Article 5 DGA are specifically tailored only to the cases of data protection, intellectual property, and secrecy. Applying the DGA to DSI would therefore require a legislative change of the DGA.

Such a legislative change is not only relevant in the case described above, where the re-use of DSI is restricted by domestic legislation and MAT in the current bilateral ABS mechanism. It is also relevant for research data other than DSI and is therefore a significant loophole in the DGA. Additionally, it would become highly relevant if a policy option with licenses is chosen for DSI.

The non-applicability of the DGA to DSI does not seem to be justified, because the DGA can apply to research data in general. Unlike in the ODD,^145^ the operational part of the DGA does not contain any special definitions of or provisions on research data held by public institutions. Therefore, *any* data held by PSBs under Article 2(17) DGA can fall under the scope of the DGA, including research data or data held by research institutions, which qualify as PSBs. Recital 12 DGA explicitly confirms that the DGA applies to research performing organizations if they are organized as PSBs or bodies governed by public law.

At the same time, Recital 12 DGA delineates or even limits^146^ the application of the DGA to research data and therefore potentially to DSI. In particular, the DGA does not apply to research data in three constellations:

First, Recital 12 excludes public research funding organizations from the scope of the DGA. Should third parties be able to approach the public funder rather than the research performing organization to request the data for re-use, this might interfere with the researchers’ incentives and means to control the timing of publication and dissemination of research data.

Second, Recital 12 excludes data which are held in the frame of research partnerships from the scope of the DGA. The wording is blurry, as it does not clearly say whether it only exempts public-private partnerships or also mere public research co-operations. However, a broad reading supports the reasonable intention of the DGA not to distract current and future incentives for establishing and conducting joint research. Especially research in genomics and related disciplines has a long history of blurred lines between public and private research – often to the benefit of innovation.^147^

Third, Recital 12 states that the DGA does not apply to ‘the exchange of data between researchers for non-commercial scientific research purposes’. This limitation appears justified on two grounds. First, the DGA should not interfere with the normal practice and culture of conducting and publishing publicly funded research. If researchers can become addressees of the DGA, while at the same time fostering research is one of the main purposes of the DGA, then the DGA runs the risk of excessive regulatory interference that might lead to unintended side-effects in daily research practice. Second, this is particularly the case for the data exchange between researchers for non-commercial research purposes, an exchange that appears to be common for DSI.^148^

Taken together, all these restrictions cut back the potential scope of application considerably. But the DGA’s prohibition of exclusive arrangements in Article 4 could be an important tool for DSI when MAT restrict re-use, also if standard MAT are chosen as a policy option for DSI and the standard MAT or licenses include restrictions to non-commercial use, for example.^149^ Article 4 DGA addresses the inherent risk of exclusive agreements to distort markets, block follow-on innovation, and thereby negatively affect social welfare by privatizing the benefits of the creation, which has been publicly funded.^150^ Accordingly, Article 4 DGA prohibits such agreements and allows them only under exceptional circumstances.^151^ Article 4 DGA is also meant to prevent such practices from being established in the future by requiring their substantial justification. This underlines the major flaw of the DGA’s narrow scope of application, which excludes DSI in such cases.

In sum, the conceptualization of the DGA is flawed in that it does not apply to MAT-restricted DSI, even though there is a substantial need for its provisions on exclusive arrangements to apply. The reason for the non-application lies in the blind eye the DGA turns to mere relative rights or unilaterally imposed restrictions that concern data, which effectively prevents its applicability to DSI from the outset. It would be up to future reform of the DGA and the ODD to plug this loophole and increase coherency. But as such a solution concerns all public sector data in general, it would not account for the specific interests relating to DSI and the underlying access and benefit-sharing rationale. Again, as in the case of the ODD, the analysis points to solutions to be found from a more holistic perspective, which would minimize the systemic tension between benefit-sharing and open access.

## V. IMPLICATIONS FOR POLICY OPTIONS FOR DIGITAL SEQUENCE INFORMATION AND THE EU OPEN DATA REGULATORY FRAMEWORK

The analysis of the ODD has revealed systemic tensions between open data principles and MAT restrictions on non-commercial re-use. It has been shown that the current scientific publishing practice and the existing database landscape are not adapted to publishing DSI with such restrictions. But if in the future a policy option for DSI is chosen, which includes MAT with restrictions on non-commercial re-use,^152^ and databases implement the possibility of such licenses, then, in its current form, it is unclear whether the ODD permits such licenses. We have argued in favor of an interpretation of the ODD that permits such licenses; however, there remains a high degree of legal uncertainty. This uncertainty concerns not only DSI but all research data with contractual restrictions of re-use. Additionally, the DGA remains toothless against exclusive agreements with commercial actors. In other words: Instead of enabling publication to maximize follow-on innovation and social welfare, the current EU open data regulatory framework together with the current bilateral approach of benefit-sharing or a future MAT license policy option favors the privatization of DSI, especially—and quite paradoxically—when the imposed restrictions apply to commercial use of DSI only. In the following, we will discuss the implications of these findings first regarding the ongoing negotiations of policy options, and then the EU open data regulatory framework.

### V.A. Towards a Specific Multilateral Benefit-Sharing Regime for Digital Sequence Information

The COP15 in Montreal in December 2022 will be of paramount importance for biodiversity, and one potential dealbreaker of the Post-2020 Global Biodiversity Framework will be the decision on how to address DSI. In the following, we will respond to the discussed policy options,^153^ considering some criteria^154^ developed for their assessment. Drawing from our analysis of DSI conditions and licenses under the ODD, our assessment will focus on option 2 of standardized MAT using standard licenses compared to the other options. Some implications can also be drawn on options 3 and 6. However, the policy options currently negotiated are not only very different from one another, but each option comes with a range of possibilities for implementation, which would greatly influence its performance under the policy goals.

Option 2 would seem a natural solution at first glance considering that the ODD promotes the use of standard licenses as well.^155^ Standard licenses have also been discussed before as a solution for DSI.^156^ Nevertheless, we caution against adopting standard licenses as a long-term solution for DSI, because the principles underlying the EU open data regulatory framework clearly point to a clash between an MAT standard license solution and other important criteria as developed for the assessment of DSI policy options.

In particular, one of the main principles of the ODD is to foster downstream innovation by mandating re-use of research data upstream for both non-commercial and commercial purposes. In contrast, the distinction between commercial and non-commercial use of genetic resources is a characteristic feature of the ABS mechanism. This upfront distinction is rooted in the Nagoya Protocol and aims to facilitate non-commercial use in order to advance basic research, including research on biodiversity.^157^ Accordingly, domestic legislation often builds upon this distinction,^158^ and the EU Nagoya Regulation reiterates the Nagoya provision.^159^ This is troublesome, since our analysis shows that this upstream differentiation between commercial and non-commercial use effectively burdens non-commercial research and ultimately favors commercial use. The reason why upstream differentiation is ineffective lies in the life cycle of genomic data: The data are supplied to the life cycle from various commercial and non-commercial sources and are then also used for non-commercial and commercial purposes. The regulatory framework for public research data in the EU has recognized this striking feature for data-related innovation and has implemented it accordingly.

Standard MAT with commercial and non-commercial licenses would be especially troublesome if such licenses were not possible within the INSDC infrastructure and DSI would instead be made publicly available in specialized repositories. This would even be the case if the genomics data infrastructure, especially the INSDC, would account for standard licenses for DSI. Building new repositories for DSI^160^ that adhere to FAIR data sharing principles^161^ requires intensive investments. It is doubtful whether researchers will take on the extra effort of using such repositories if they are not specifically interested in certain DSI. It has been noted that trust in the quality of the data and the curation of the database are essential for researchers to use data generated by other researchers.^162^ But smaller repositories with restrictions on re-use could create ‘data silos’ and thereby hinder research and innovation.^163^ Just as jurisdiction shopping should be avoided when choosing a policy option,^164^ so should ‘data forum shopping’.

Even if INSDC databases were to adopt multiple standard licenses distinguishing between commercial and non-commercial purposes to account for benefit-sharing obligations, these licenses would incentivize using data only for commercial use and without the need for benefit-sharing from the genomics data corpus. For good reason, open access and the central genomic data infrastructure of the INSDC have evolved within the scientific genomics community. To make sense of genomic data, the comparison of a vast amount of other sequences is utterly important. Therefore, the publication of DSI with non-commercial restrictions could effectively exclude DSI from the genomic data life cycle, just as it is already excluded if no sharing is possible. While it would seem at first glance that a policy option using standard licenses would perform well under criterion 11 to enable a distinction between commercial and non-commercial use of DSI,^165^ this result is detrimental to the criterion that the policy option should not hinder research and innovation.^166^ Standard licenses would also not deliver predictable monetary benefits.^167^ Perhaps even more dire, it would hinder the very much needed research on biodiversity in megadiverse countries.

The picture would be different if under policy option 2 a single MAT were to be negotiated and implemented into the INSDC. A single MAT would, however, require that benefit-sharing is based on the whole corpus of the INSDC, and no distinction would be made between commercial and non-commercial use.^168^ Such a solution would avoid the creation of data silos or data forum shopping, and users would not be incentivized to use data without benefit-sharing obligations. A single MAT would be well aligned with open data principles, as it would not distinguish between commercial and non-commercial use. As becomes apparent, policy option 2 can be implemented in many different ways. While standard MAT on a national level would be very close to the current uncertain legal status of DSI with high transaction costs, a single international MAT implemented in the INSDC is much closer to policy option 3.

Given this heterogeneous landscape of policy options, the analysis of the open data principles guides us to one conclusion: Any policy option chosen should not distinguish between commercial and non-commercial use upstream, but, if at all necessary, later downstream. From the range of the stated policy options (and supposing that options 4 and 5 would not be grounds for a political compromise due to lack of (monetary) benefit-sharing), this would point towards a solution within a multilateral mechanism. Such a mechanism could be option 3, more specifically option 3.2 of other payments and contributions such as from the storage or analysis of sequences, a micro-levy on laboratory equipment, or biodiversity bonds.^169^ From an open data point of view, however, we advise against a policy option generating benefits from payment for access to DSI.^170^ Payments for access would run counter to open data principles^171^ and create entry barriers for start-ups and SMEs.^172^ A recent academic proposal strongly argued for a multilateral benefit-sharing mechanism, which is decoupled from access to genetic resources.^173^ The authors propose either a monetary mechanism upstream or – and this would be rather in line with our analysis – downstream or outside the DSI life cycle.^174^ The proposal is especially compelling in that it proposes to base benefit-sharing upon the entire global dataset and not individual sequences.^175^ Such a solution could also be realized within option 2, but only if a single MAT were implemented in the INSDC.

Another option consistent with our findings could be option 6, according to which one per cent on retail sales of genetic resources would be charged as a levy. The recommendation of the Open-ended Working Group included a proposal for the establishment of a multilateral benefit-sharing mechanism,^176^ envisaging that:

1 per cent of the retail price of all commercial income resulting from all utilization of genetic resources, traditional knowledge associated with genetic resources or digital sequence information on genetic resources is shared through the multilateral benefit-sharing mechanism to support the conservation and sustainable use of biological diversity, unless such benefits are otherwise being shared on mutually agreed terms established under the bilateral system.

The proposal remains rather vague, and just like the other policy options, its effectiveness will depend on its concrete implementation. However, some implications can be drawn from the open data perspective. While the proposal includes a distinction between commercial and non-commercial use, this distinction would occur downstream and therefore likely not hinder research and innovation but generate predictable monetary benefits, which could be redistributed.^177^ However, the one per cent amount of the tax or levy could certainly be disputed. Caution should also be exercised with regard to establishing such a mechanism in parallel to a bilateral system, which could add complexity and uncertainty to an already complex and uncertain ABS mechanism.

### V.B. Fixing the EU Open Data Regulatory Framework

In case policy option 2 were to be chosen and implemented with licenses distinguishing between commercial and non-commercial use, the ODD would need to be revised to explicitly allow for such a distinction. Although we have argued for an interpretation of the current ODD that permits such a distinction, an amendment of the ODD is necessary to provide legal certainty for researchers. The use case for such an amendment goes beyond DSI and pertains to all research data, which cannot be made publicly available without restrictions due to contractual obligations. For example, there are other specialized repositories using CC licenses including CC-NC.^178^ While the principle of the ODD not to distinguish between commercial and non-commercial re-use should not be undermined, it seems to be in line with the principle of ‘as open as possible, as closed as necessary’ to allow such restrictions in certain cases. We have argued above that such a case should be contractual obligations to third parties. A future revision of the ODD should include a clarification that in case of contractual restrictions imposed by a third party, a non-commercial condition for the re-use of research data is allowed under the ODD.

For cases in which the ODD does not apply, eg if research data is not made publicly available, however, publicly funded research could still be shared exclusively and hence be privatized. It is therefore crucial that such exclusive arrangements are subject to the scope of the DGA. Accordingly, the DGA should be amended in such a way that it would also cover data, which cannot be published due to contractual restrictions between the PSB and a third party. As a consequence, the DGA could apply to research data within the meaning of the ODD, including DSI, even if they are not made publicly available. However, in this regard it is important to keep the exemption in the DGA concerning the sharing of research data between researchers for non-commercial use only. Ideally, the ODD and the DGA would be revised jointly and recast in one regulation to streamline the open data regulatory framework.^179^

## VI. CONCLUSION

Over the past 30 years, efforts to conserve biodiversity and to share fairly and equitably the benefits arising from the utilization of genetic resources have led to the establishment of a complex legal framework of access and benefit-sharing. At the same time, following the Human Genome Project and benefitting from its strong open data culture, research methods on genomics have significantly advanced, and genomic data have grown exponentially. This advance has led to the question of whether and how the access and benefit-sharing legal framework could be extended to DSI, which is currently negotiated as part of the Post-2020 Global Biodiversity Framework. Meanwhile, the EU open data legislative framework of the ODD and the DGA already covers research data including DSI. An analysis of the ODD and the DGA reveals systemic tensions between open data principles and MAT license restrictions on non-commercial use. On the one hand, the ODD’s rationale is to make publicly funded data upstream re-usable to allow follow-on innovation downstream. On the other hand, the access and benefit-sharing mechanism follows a rationale where the use of genetic resources—and in some countries DSI—is regulated to share equitably the benefits from resulting downstream innovation. The analysis first reveals shortcomings of both the ODD and the DGA, which are relevant beyond their application to DSI. These shortcomings should be addressed in future revisions by clarifying the application of the ODD to cases in which contractual obligations with third parties impose restrictions on the re-use of research data, and by including these cases in the scope of application of the DGA. Second, drawing from this analysis, we advise against a policy option for DSI, which distinguishes between commercial and non-commercial use upstream, arguing instead for a policy option, which imposes benefit-sharing obligations further downstream or outside of the DSI life cycle. The negotiations at the COP15 in Montreal in December 2022 are of paramount importance not only for the conservation of biodiversity and the fair and equitable sharing of benefits from genetic resources, but also for the openness of research data.

## Footnotes

1 The Convention on Biological Diversity, 5 June 1992, 1760 U.N.T.S. 79.

2 See Patrick Greenfield, *Biopiracy Row at UN Talks in Geneva threatens Global Deal to Save Nature*, The Guardian, 22 March 2022; Erik Sokstad, ‘*It’s a Thorny Issue’ Why a Fight over DNA Data Imperils a Global Conservation Pact,*Science, 5 April 2022.

3 For an overview, see Margo Bagley, et al., *Fact-finding Study on How Domestic Measures Address Benefit-sharing Arising from Commercial and Non-commercial Use of Digital Sequence Information on Genetic Resources and Address the Use of Digital Sequence Information on Genetic Resources for Research and Development,* U.N. Doc. CBD/DSI/AHTEG/2020/1/5, at 10 (29 January 2020).

4 See The Open-ended Working Group on the Post-2020 Global Biodiversity Framework, *Digital Sequence Information on Genetic Resources: Addendum Note by the Executive Secretary,* U.N. Doc. CBD/WG2020/3/4/Add.1, Annex I (26 November 2021).

5 Directive 2019/1024 of the European Parliament and of the Council of 20 June 2019 on open data and the re-use of public sector information, OJ L 172/56, 26 June 2019.

6 Regulation 2022/868 of the European Parliament and of the Council of 30 May 2022 on European data governance and amending Regulation (EU) 2018/1724 (Data Governance Act), OJ L 152/1, 3 June 2022.

7 See eg Dominique Foray, The Economics of Knowledge (2004); Brett M. Frischmann and Mark A. Lemley, *Spillovers*, 107 Columbia Law Review 257 (2007).

8 See the publication of the public consortium: Eric S. Lander, et al*., Initial Sequencing and Analysis of the Human Genome*, 409 Nature 860 (2001); The private initiative J. Craig Venter, et al., *The Sequence of the Human Genome,* 291 Science 1304 (2001).

9 See Kathryn Maxson Jones, et al., *The Bermuda Triangle: The Pragmatics, Policies, and Principles for Data Sharing in the History of the Human Genome Project,* 51 J Hist Biol 693, 778 (2018).

10 See Eliot Marshall, *Genome Researchers Take the Pledge,* 272 Science 477 (1996).

11 This meant that the private company Celera Genomics used the publicly funded sequence data in the race for the first assembled human genome, but not vice versa, which turned out to accelerate the sequencing efforts of the public consortium, too, contributing to the timely publication of the Human Genome Project, see Jones, *supra* note 9 at 764–769.

12 See Jerome H. Reichman, Paul F. Uhlir & Tom Dedeurwaerdere, Governing digitally integrated genetic resources, data, and literature: global intellectual property strategies for a redesigned microbial research commons 412 (2016).

13 Genomics is the research of genomes, a term originally created in the 1980s to refer to the sequencing of genomes, mapping genes within the genome, and the technology used, see S. Kuska, *Beer, Bethesda, and Biology: How ‘Genomics’ Came into Being,* 90 J Natl Cancer Inst. 93 (1998).

14 See Youngseek Kim, *Data Sharing by Biologists: A Comparative Study of Genome Sequence Data and Lab Experiment Data*, 44 Library & Information Science Research, art. no. 101139 (2022); Fábio C.P. Navarro, et al., *Genomics and Data Science: An Application within an Umbrella*, 20 Genome Biol, art. no. 109, at 7–8 (2019); Ryan P. Womack, *Research Data in Core Journals in Biology, Chemistry, Mathematics, and Physics*, 10 Plos one, art. no. e0143460 (2015).

15 See Sriram Subramaniam, Gerard J. Kleywegt, *A Paradigm Shift in Structural Biology*, 19 Nature Methods 20 (2022); And Vivien Marx, *Method of the Year*, 19 nature methods 5 (2022).

16 See John Jumper, et al.*, Highly Accurate Protein Structure Prediction with AlphaFold*, 596 Nature, 583, 585 and Methods (2021).

17 See Peter W. Harrison, et al., *The COVID-19 Data Portal: Accelerating SARS-CoV-2 and COVID-19 Research through Rapid Open Access Data Sharing,* 49 Nucleic Acids Research, 2 July 2021, at W619 (2021).

18 Regarding the US, see Jalayne J. Arias, et al., *The Growth and Gaps of Genetic Data Sharing Policies in the United States,* 2 Journal of Law and the Biosciences 56 (2015); regarding the EU, see the analysis below (III).

19 Article 1 CBD.

20 Nagoya Protocol on Access to Genetic Resources and the Fair and Equitable Sharing of Benefits Arising from their Utilization to the Convention on Biological Diversity, *Tenth Meeting of the Parties to the Convention on Biological Diversity*, U.N. Doc. UNEP/CBD/COP/DEC/X/1 (29 October 2010).

21 Article 5(1), Article 6(1) Nagoya Protocol.

22 Canada and Russia are only party to the CBD; the US is neither party to the CBD nor the Nagoya Protocol; see the list of parties at https://www.cbd.int/information/parties.shtml#tab=2 (accessed 10 September 2022).

23 Regulation (EU) No 511/2014 of the European Parliament and of the Council of 16 April 2014 on compliance measures for users from the Nagoya Protocol on Access to Genetic Resources and the Fair and Equitable Sharing of Benefits Arising from their Utilization in the Union (Text with EEA relevance), OJ L 150/59, 20 May 2014.

24 See Article 4 Nagoya Regulation.

25 For an overview, see Brendan Coolsaet, *Conclusion. Comparing Access and Benefit-Sharing in Europe*, *in*Implementing the Nagoya Protocol 363 (Brendan Coolsaet et al. eds., 2015).

26 Conference of the Parties to the CBD, U.N. Doc. COP 2 Decision II/11, para. 2; Recital 19 Nagoya Regulation.

27 See Article 2(2), Article 4(4), Recital 12 Nagoya Regulation.

28 National Human Genome Research Institute, *DNA Sequencing Costs: Data*, https://www.genome.gov/about-genomics/fact-sheets/DNA-Sequencing-Costs-Data (accessed 10 September 2022).

29 This question is not only debated between the parties to the CBD and the Nagoya Protocol, but also in the related international fora of the International Treaty on Plant Genetic Resources for Food and Agriculture, the Pandemic Influenza Preparedness Framework, and the Convention on the Law of the Sea.

30 See Bagley, *supra* note 3, at 10.

31 Section 1 under ‘genetic resource’ (b) National Environmental Management Biodiversity Act, Act 10 of 2004, as amended by the National Environmental Management Laws Amendment Act, Act 14 of 2013; see also African Group of Negotiators on Biodiversity-Ad Hoc Group on Digital Sequence Information, *Digital Sequence Information on Genetic Resources: Submission of Views and Information on Terminology, Scope, and Domestic Measures on Access and Benefit Sharing*, 6 (2019); see Bagley, *supra* note 3, at 13 (footnote 71) and 18.

32 See Sec. 81 National Environmental Management Biodiversity Act, Act 10 of 2004, as amended by the National Environmental Management Laws Amendment Act, Act 14 of 2013; see also Sec. 1(1), 80(2) for the definition of ‘indigenous biological resource’ and ‘bioprospecting’ in the same Act.

33 See Sec. 80(1) lit. c, 83 National Environmental Management Biodiversity Act, Act 10 of 2004, as amended by the National Environmental Management Laws Amendment Act, Act 14 of 2013, see also Sec. 1(1) for the definition of ‘bioprospecting’ in the same Act.

34 See Sec. 13(2) lit. f Regulations on Bio-prospecting, Access and Benefit-Sharing of 2008.

35 See Sec. 9 of the Material Transfer Agreement of the National Model Contractual Clause of South Africa as available at the Access and Benefit-Sharing Clearing House, https://absch.cbd.int/en/countries/ZA/NMCC (accessed 10 September 2022). It should be noted that the terms ‘Material Transfer Agreement’ and ‘Mutually Agreed Terms’ are sometimes used synonymously, depending on the national legislation, see Bagley, *supra* note 3, at 23. Within this article, we use the term ‘Mutually Agreed Terms’ for all contractual terms regarding access and benefit-sharing, which is in line with the terminology used by the EU Nagoya Regulation.

36 For an overview regarding DSI, see Bagley, *supra* note 3; see also the terms and conditions of a Registered Collection of the Nagoya Regulation, the German Collection of Microorganisms and Cell Cultures, §10, https://www.dsmz.de/terms (accessed 10 September 2022). Regarding the distinction in ABS in general, see Caroline von Kries & Gerd Winter, *Defining commercial and non-commercial research and development under the Nagoya Protocol and in other contexts*, *in*Research and Development on Genetic Resources 60, 65 (Evanson Chege Kamau, Gerd Winter, & Peter-Tobias Stoll eds., 2015).

37 See von Kries and Winter, *supra* note 36, at 65.

38 See European Commission Notice Guidance document on the scope of application and core obligations of Regulation (EU) No 511/2014 of the European Parliament and of the Council on the compliance measures for users from the Nagoya Protocol on Access to Genetic Resources and the Fair and Equitable Sharing of Benefits Arising from their Utilisation in the Union, OJ C 313/01, 27.8.2016, at 2.3.5.

39 For an overview, see Bagley, *supra* note 3, at 18.

40 See Lilian Massini Mozini, *Brazilian Biodiversity Law: Challenges and Opportunities for Industries and Research Institutions*, *in*Global Transformations in the Use of Biodiversity for Research and Development 69, 76 f. (Evanson Chege Kamau ed., 2022).

41 See The Open-ended Working Group on the Post-2020 Global Biodiversity Framework, U.N. Doc. CBD/WG2020/3/4/Add.1, *supra* note 4, at 7–9.

42 It was not present in the original typology of policy options, see CBD Secretariat, Policy Options for Access and Benefit-sharing and Digital Sequence Information, 3 Figure 1, April 2021, http://www.cbd.int/abs/DSI-webinar/DSIPolicyOptions2021.pdf (accessed 10 September 2022).

43
*Id.*

44 Article 10 Nagoya Protocol sets out the possibility to establish a global multilateral benefit-sharing mechanism for transboundary situations or if prior informed consent cannot be obtained. It was introduced as a political compromise in the final hours of the negotiations of the Nagoya Protocol and therefore remains rather vague, see Elisa Morgera, Elsa Tsioumani and Mathias Buck, Unraveling the Nagoya Protocol 198 (2014).

45 See Open-Ended Working Group on the Post-2020 Global Biodiversity Framework, *Digital Sequence Information on Genetic Resources*, U.N. Doc. CBD/WG2020/4/L.3, 5 (26 June 2022) referring to Open-Ended Working Group on the Post-2020 Global Biodiversity Framework, *Co-Leads’ Report on the Work of the Informal Co-Chairs’ Advisory Group on Digital Sequence Information on Genetic Resources*, U.N. Doc. CBD/WG2020/3/INF/8, paras. 40 f. (18 November 2021).

46 See Wael Houssen, et al., *Digital Sequence Information on Genetic Resources: Concept, Scope and Current Use*, U.N. Doc. CBD/DSI/AHTEG/2020/1/3, 10 (29 January 2020).

47 This flow of genetic information is part of the central dogma of molecular biology by Francis Crick, see Francis H.C. Crick, *The Symposia of the Society for Experimental Biology*, 12 Cambridge University Press, 138, 153 (1958); Francis Crick, *Central Dogma of Molecular Biology,* 227 Nature 561, 562 (1970).

48 See The Open-ended Working Group on the Post-2020 Global Biodiversity Framework, U.N. Doc. CBD/WG2020/4/L.3, *supra* note 45, at 2–3.

49 Member States could, of course, adopt national legislation, as happened, for example, in France, see Sara Gobbato, *Open Science and the Reuse of Publicly Funded Research Data in the New Directive (EU) 2019/1024,* 2 Journal of Ethics and Legal Technologies 145, 147–148 (2020).

50 The ODD uses the general term ‘documents’, see below III.C.1.

51 Directive 2013/37/EU of the European Parliament and of the Council of 26 June 2013 amending Directive 2003/98/EC on the re-use of public sector information, OJ L 175/1, 27 June 2013.

52 See Article 1(2) lit. e PSI Directive.

53 See Heiko Richter*, Open Science and Public Sector Information – Reconsidering the Exemption for Educational and Research Establishments under the Directive on Re-use of Public Sector Information,* 2018 JIPITEC 51.

54
*Id.* at 53.

55 See Article 1, Recital 3 ODD.

56 Recital 27 ODD.

57 Recital 3 ODD.

58 In general, access to public sector information remains within the competencies of the Members States. See Mireille van Eechoud and Katleen Janssen, *Rights of Access to Public Sector Information*, 6 Masaryk University Journal of Law and Technology 471, 476 (2012).

59 See Recital 27 ODD.

60 Most of them incidentally with a genomics background, see Mark D. Wilkinson et al., *The FAIR Guiding Principles for Scientific Data Management and Stewardship*, 3 Scientific Data, art. no. 160018 (2016).

61 Article 2(6) ODD.

62
Heiko Richter, Informationsweiterverwendungsgesetz: Kommentar, § 2, at no. 69 (2018).

63 For example, one could consider documents in a digital form to include in vivo or in silico DNA storage mediums. For an overview of DNA storage methods, see Luis Ceze, et al., *Molecular Digital Data Storage using DNA,* 20 Nature Reviews Genetics 456 (2019).

64 Recital 27 ODD.

65 Provided that the other requirements are met.

66 Although RNA is usually transcribed into cDNA for sequencing.

67 See Richter*, supra* note 53, at 56 (referring to gene sequences); Judith Zimmermann, *Zum Potenzial des europäischen Weiterverwendungsrechts für die Erforschung der Biodiversität*, 2021 Zeitschrift für Umweltrecht 84, 91 (referring to digital sequence data).

68 Navarro*, supra* note 14, at 7 f.

69 See The Open-ended Working Group on the Post-2020 Global Biodiversity Framework, CBD/WG2020/4/L.3*supra* note 45, at 2 f.; Houssen, *supra* note 46, at 32.

70 See Article 1(2) lit. h and 1(4) ODD; Regarding challenges in genomic data sharing and privacy, see Arias, *supra* note 18, at 56–68; Ellen Wright Clayton, et al., *The Law of Genetic Privacy: Applications, Implications, and Limitations*, 6 Journal of Law and the Biosciences 1 (2019). Regarding human genomic data and data protection in the EU, see Kärt Pormeister, *Genetic Research and Applicable Law: The Intra-EU Conflict of Laws as a Regulatory Challenge to Cross-Border Genetic Research*, 5 Journal of Law and the Biosciences 706 (2018).

71 Recital 30 ODD.

72 Article 2(9) ODD.

73 See Gobbato, *supra* note 49, at 152; Zimmermann*, supra* note 67, at 87.

74 See Article 1(2) lit. c and Article 10(2) ODD. This exemption ‘refers to copyright and related rights only, including sui generis forms of protection’, see Recital 54 ODD.

75 See Richter*, supra* note 53, at 56.

76 See European Commission Staff Working Document Impact Assessment, Proposal for a Directive of the European Parliament and of the Council on the re-use of public sector information, SWD(2018) 128 final, 38 f.

77 Similarly, Gobbato, *supra* note 49, at 152.

78 See Richter*, supra* note 53, at 59.

79 Tom Dedeurwaerdere, Paolo Melindi-Ghidi & Arianna Broggiato, *Global scientific research commons under the Nagoya Protocol: Towards a collaborative economy model for the sharing of basic research assets*, 55 Environ Sci Policy 1, 4 (2016).

80 See for example here: Guibing Hu, et al., *Two Divergent Haplotypes from a Highly Heterozygous Lychee Genome Suggest Independent Domestication Events for Early and Late-maturing Cultivars*, 54 Nat Genet 73 (2022).

81 The code may fall under the rules of the ODD if the Member States have chosen to do so.

82 See also Gobbato, *supra* note 49, at 152: research data as supporting information.

83 See Zimmermann, *supra* note 67, at 87.

84 Article 10(2) ODD.

85 See Heiko Richter*,* Stellungnahme zum Entwurf eines Gesetzes zur Änderung des E-Government-Gesetzes und zur Einführung des Gesetzes für die Nutzung von Daten des öffentlichen Sektors vom 17.12.2020, at 14, https://www.ip.mpg.de/fileadmin/ipmpg/content/stellungnahmen/2021-01-12_Richter_Stellungnahme_Open_Data_Gesetz.pdf (accessed 10 September 2022).

86 See eg the members of the Alliance of Science Organizations in Germany.

87 Public-private partnerships also appear to be common in companies using and generating DSI, see Fabian Rohden, et al., *Combined Study on Digital Sequence Information (DSI) in Public and Private Databases and Traceability*, U.N. Doc. CBD/DSI/AHTEG/2020/1/4, 39 and 78 with case studies 5 and 6 (31 January 2020).

88 See Article 10(2) ODD. In Germany, this has been implemented as an exclusion, see § 2 Abs. 2 Nr. 3 DNG.

89 Article 10(2) ODD.

90 Recital 28 ODD. Initially, a policy option was considered where all research results including publications were to be made open access, see European Commission, *supra* note 38, at 30.

91 See eg the annual database issue of the Nucleic Acids Research Journal: Daniel J Rigden and Xosé M Fernández, *The 2022 Nucleic Acids Research Database Issue and the Online Molecular Biology Database Collection,* 50 Nucleic Acids Research, D1–D10 (2022).

92 Ensembl, *SARS-CoV-2: Reference Genome,*https://covid-19.ensembl.org (accessed 10 September 2022).

93 Navarro*, supra* note 14, at 7 f.

94 See possible repositories for Scientific Data, *Data Repository Guidance*, https://www.nature.com/sdata/policies/repositories (accessed 10 September 2022); and Science, *Science Journals: Editorial Policies,*https://www.science.org/content/page/science-journals-editorial-policies#data-and-code-deposition (accessed 10 September 2022). See also Rohden, *supra* note 87, at 23 f.

95 See Rohden, *supra* note 87, at 18 ff.

96 Article 10(2) ODD.

97 Though universities as research performing organisations are an important use case, see Article 2(4) ODD.

98 Zimmermann, *supra* note 67, at 87; the term research organisation is thus defined much more broadly than in the EU Digital Single Market Directive, see Article 2(1) Digital Single Market Directive.

99 These provisions regulate the re-use of documents in general, but also apply to research data, Article 10(2) ODD.

100 See Article 10(2) ODD for research data, Article 3 ODD for other public sector data and information.

101 See Reichman, Uhlir, and Dedeurwaerdere, *supra* note 12, at 376, 378 f., 401 f.

102 Standard licences are defined as ‘a set of predefined re-use conditions in a digital format, preferably compatible with standardised public licences available online’, see Article 2(5) ODD, Recital 44 ODD.

103 E.g. under a BY-NC licence, only non-commercial use is allowed, and the author must be named.

104 See European Commission, *EU Grants: AGA Annotated Model Grant Agreement, EU Funding Programmes 2021–2027*, 152 (2021), https://ec.europa.eu/info/funding-tenders/opportunities/docs/2021-2027/common/guidance/aga_en.pdf (accessed 10 September 2022).

105 Cf. Reichman, Uhlir, and Dedeurwaerdere, *supra* note 12, at 402 f.; Dedeurwaerdere, Melindi-Ghidi, and Broggiato, *supra* note 79, at 9; and specifically regarding the role of the state Jorge L. Contreras, *Leviathan in the Commons: Biomedical Data and the State*, *in*Governing Medical Knowledge Commons 19 (Katherine J. Strandburg, Brett M. Frischmann, & Michael J. Madison eds., 2017).

106 Research data generated from EU grants must be made available ‘under the latest available version of the Creative Commons Attribution International Public License (CC BY) or Creative Commons Public Domain Dedication (CC0) or a licence with equivalent rights’, see European Commission, supra note 104, at 152. The attribution module is consistent with good scientific practice and with Article 8(1) ODD, see Recital 44 ODD.

107 See Richter, *supra* note 62, at § 4, no. 84; Recital 19 PSI Directive. On the applicability of CC licensing to public sector information, see Mireille van Eechoud and Brenda van der Wal, *Creative Commons Licensing for Public Sector Information*, IViR (2008).

108 This was already the case before the recast as the ODD: Richter, *supra* note 62, at § 4 Rn. 83; *Andreas Wiebe & Elisabeth Ahnefeld, Zugang zu und Verwertung von Informationen der öffentlichen Hand – Teil II*, 2015 Computer und Recht 199, 207.

109 See above section III.C.5.

110 E.g. NCBI, which includes GenBank, does not accept restrictions on re-use or re-distribution, see U.S. National Library of Medicine, *NCBI Website and Data Usage Policies and Disclaimers*, https://www.ncbi.nlm.nih.gov/home/about/policies/ (accessed 10 September 2022).

111 For an overview, see Rodrigo Sara et al., *Open access: a technical assessment for the debate on benefit-sharing and digital sequence information*, at 18 f. (2022).

112 This issue is also highlighted regarding South Africa by the submission of the African Group of Negotiators on Biodiversity-Ad Hoc Group on Digital Sequence Information, *supra* note 31, at 6.

113 Recital 28 ODD.

114 Deloitte, *Study to Support the Review of Directive 2003/98/EC on the Re-use of Public Sector Information*, European Commission, 242 (2018).

115 See *Open for Business*, 4 Sci Data, art. no 170058 (2017).

116 As has been proposed as a possible solution for DSI: Kaspar Sollberger, *Digitale Sequenzinformationen und das Nagoya-Protokoll: Rechtliches Kurzgutachten im Auftrag des Bundesamts für Umwelt (BAFU)*, at 18 (2018).

117 See The Open-ended Working Group on the Post-2020 Global Biodiversity Framework, U.N. Doc. CBD/WG2020/3/4/Add.1, *supra* note 4, at 8.

118
*Id.* at 8.

119
*Id.* 11.

120 Argument: Article 1(2) ODD.

121 It thus aims to strike a balance in the diffusion-innovation dilemma, a classical problem in the science commons, see Robert Cook-Deegan & Tom Dedeurwaerdere, *The science commons in life science research: structure, function, and value of access to genetic diversity*, 58 Int Social Science J 299, 310 (2006).

122 Documents for which their parties hold intellectual property rights are excluded, Article 1(2) lit. c ODD; with regard to research data, pre-existing intellectual property rights are to be taken into account, Article 10(2) ODD; see also Recital 54 ODD, which states that the ODD ‘does not apply to documents covered by industrial property rights, such as patents and registered designs and trade marks’.

123 See Article 10(2) ODD. In Germany, this provision has been implemented as an exception, see § 2(2) No 3 Datennutzungsgesetz.

124 See Recital 28 ODD. Regarding trade secret protection in genomics as a response to Mayo Collaborative Services v. Prometheus Laboratories, Inc., 566 U.S. 66 (2012) and Association for Molecular Pathology v. Myriad Genetics, Inc., 569 U.S. 576 (2013), see Christi J Guerrini, et al., *Constraints on Gene Patent Protection Fuel Secrecy Concerns: A Qualitative Study*, 4 Journal of Law and the Biosciences 542 (2017).

125 See Claudia Seitz*, Digital Sequence Information – Legal Questions for Patent, Copyright, Trade Secret Protection and Sharing of Genomic Sequencing Data*, 482 IOP Conf. Ser.: Earth Environ. Sci. 4 (2020).

126 See Article 1(4) ODD.

127 See Article 1(2) lit. h ODD.

128 See Recital 44 ODD.

129 See Arias, *supra* note 18, at 64.

130 Cf. Sara, *supra* note 111, at 18 f.

131 Article 10(1) ODD.

132 See also Recital 16 ODD.

133 See von Kries and Winter, *supra* note 36, at 65, 67.

134 The Open-ended Working Group on the Post-2020 Global Biodiversity Framework, U.N. Doc. CBD/WG2020/3/4/Add.1, *supra* note 4, at 10.

135
Reichman, Uhlir, and Dedeurwaerdere, *supra* note 12, at 366; von Kries and Winter, *supra* note 36, at 66.

136 See Article 3(1), Recital 6 DGA. Unlike the ODD, the DGA only applies to data held by PSBs and not by public undertakings, see Article 3(2) lit. a DGA.

137 See Recital 6 DGA.

138 See Article 4 et seq. DGA.

139 See Article 4 DGA.

140 See Article 6 DGA.

141 See Article 1(2), Recital 11 DGA.

142 Commission Staff Working Document Impact Assessment Report, Proposal for a Regulation of the European Parliament and of the Council on European data governance (Data Governance Act), SWD (2020) 295 final, p. 25.

143 See analysis in SMART 2020/694 D2, 145 ff., also on particular case studies.

144 Even if one would argue that DSI can qualify for *sui generis* right protection under the EU Directive 96/9/EC (Database Directive), Article 5(7) DGA does not allow the PSBs (meaning here: the research institution who performed the sequencing) to exercise this right to prevent the re-use of data or to restrict re-use beyond the limits set by the DGA.

145 Unlike special rules in the ODD, eg Article 1(1) lit. c, Article 1(2) lit. l, Article 10 ODD.

146 The limitations are significant, and it appears questionable that they are only mentioned in the recitals and not the operational text to the Regulation.

147 See Cook-Deegan and Dedeurwaerdere, *supra* note 121 at 303–306 (although the authors caution against placing too much trust in the free market).

148 See Elizabeth Karger, *Study on the use of digital sequence information on genetic resources in Germany*, at 32 Figure 11 (2018). Informal exchanges also seem to be common in other areas of the life sciences such as microbial research, see Tom Dedeurwaerdere, *Global microbial commons: institutional challenges for the global exchange and distribution of microorganisms in the life sciences*, 161 Res Microbiol 414, 414 f. (2010).

149 See the study regarding in-house databases of nucleotide sequence data, which consist mostly of data obtained from public databases, data generated by the company itself, but also data obtained through third parties: Rohden, *supra* note 87, at 36 and 39, and 78 with case studies 5 and 6. See also case study 4 of a company in plant breeding, whose in-house database contains nucleotide sequence data from public databases, from in-house sequencing, and provided by customers including academic institutions.

150 For published DSI, Article 12 ODD contains a similar prohibition. However, this appears of limited practical relevance because such data have to meet all requirements of Article 10(2), see also Article 1(2) lit. l ODD; Zimmermann*, supra* note 67, at 88.

151 See Article 4(2) DGA.

152 As is a possibility, see The Open-ended Working Group on the Post-2020 Global Biodiversity Framework, U.N. Doc. CBD/WG2020/3/4/Add.1, *supra* note 4, at 8.

153 For an overview of the policy options, see Section II.A.3.

154 See The Open-ended Working Group on the Post-2020 Global Biodiversity Framework, U.N. Doc. CBD/WG2020/3/4/Add.1, *supra* note 4, at 10 f.

155 See Article 8 and Recital 44 ODD.

156 See The Open-ended Working Group on the Post-2020 Global Biodiversity Framework, U.N. Doc. CBD/WG2020/3/4/Add.1, *supra* note 4, at. 7. See also Paul Oldham, *Digital Sequence Information – Technical Aspects*, at 34–36 (2020); As part of a possible sui generis approach for DSI inspired by copyright: Elisa Morgera, et al., *Study for the European Commission on ‘Possible Ways to Address Digital Sequence Information – Legal and Policy Aspects*, at 31 (2020); Amber Hartman Scholz, et al., *Finding Compromise on ABS & DSI in the CBD: Requirements & Policy Ideas from a Scientific Perspective*, WiLDSI white paper (WiLDSI is an acronym for Wissenschaftliche Lösungsansätze für Digitale Sequenzinformation (Scientific approaches for digital sequence information), a project funded by the German Federal Ministry of Education and Research), at 26–30 (2020).

157 See Article 8(a) Nagoya Protocol.

158 South Africa distinguishes in its access permit and utilisation obligations in MAT between commercial and non-commercial purposes, see *infra* section II.B.2. For other countries, see also Bagley, *supra* note 3, eg at 37 regarding Australia, at 24 regarding Belarus, at 43 regarding Costa Rica. See also von Kries and Winter, *supra* note 36, at 65.

159 See Recital 18 Nagoya Regulation.

160 As has been proposed as a possible solution for DSI: see Sollberger, *supra* note 116, at 18.

161 See Article 10(1) ODD.

162
Reichman, Uhlir, and Dedeurwaerdere, *supra* note 12 at 436.

163 See Sara, *supra* note 111, at Table 1.

164 See Criterion 16 The Open-ended Working Group on the Post-2020 Global Biodiversity Framework, U.N. Doc. CBD/WG2020/3/4/Add.1, *supra* note 4, at 11.

165 See *Id.* at 11.

166 Criterion 4 *Id.* at 10.

167 Criterion 1 *Id.* at 10.

168 Cf. Amber Hartman Scholz, et al., *Multilateral Benefit-sharing from Digital Sequence Information will Support both Science and Biodiversity Conservation*, 13 Nat Commun, art. no. 1086, at 3 (2022).

169 See The Open-ended Working Group on the Post-2020 Global Biodiversity Framework, U.N. Doc. CBD/WG2020/3/4/Add.1, *supra* note 4, at 8.

170 See *Id*. at 8.

171 See Article 6(6) lit. b ODD regarding research data.

172 See Recital 36 ODD.

173 See Scholz, *supra* note 168. Regarding the delinking of access and benefit-sharing and various upstream and downstream models, see also Gerd Winter, *Problems and Solutions of Access to Genetic Resources and Benefit Sharing: A Theoretical Perspective - Part II*, 17 LEAD, art. no. 1706 (2021).

174 See Scholz, *supra* note 168, at 3

175 See *Id.* at 3. It should be noted that the paper uses the term DSI differently than in this article.

176 The Open-ended Working Group on the Post-2020 Global Biodiversity Framework, U.N. Doc. CBD/WG2020/4/L.3, *supra* note 45.

177 See The Open-ended Working Group on the Post-2020 Global Biodiversity Framework, U.N. Doc. CBD/WG2020/3/4/Add.1, *supra* note 4, at 10 f., criteria 1, 4, and 11.

178 One example is a repository of UK biodiversity data, the NBN atlas, although in the UK, see https://docs.nbnatlas.org/data-licenses/ (accessed 10 September 2022); see also Sara, *supra* note 111, at 19.

179 According to Article 18 ODD, the Commission will evaluate the Directive no sooner than 17 July 2025.

